# SiO_2_ nanosphere coated tough catheter with superhydrophobic surface for improving the antibacteria and hemocompatibility

**DOI:** 10.3389/fbioe.2022.1067139

**Published:** 2023-01-10

**Authors:** Weixing Zhang, Juan Du, Tonghe Zhu, Ruilan Wang

**Affiliations:** ^1^ Department of Critical Care Medicine, School of Medicine, Shanghai General Hospital, Shanghai Jiao Tong University, Shanghai, China; ^2^ School of Chemistry and Chemical Engineering, Shanghai Engineering Research Center of Pharmaceutical Intelligent Equipment, Shanghai Frontiers Science Research Center for Druggability of Cardiovascular Non-coding RNA, Institute for Frontier Medical Technology, Shanghai University of Engineering Science, Shanghai, China

**Keywords:** superhydrophobic, SiO_2_ nanosphere, catheter, antibacterial, blood compatibility

## Abstract

Catheter infection is the most common complication after vascular catheter placement, which seriously threatens the survival of critically ill patients. Although catheters with antibacterial drug coatings have been used, catheter infections have not been effectively resolved. In this research, a SiO_2_ nanosphere-coated PTFE catheter (PTFE-SiO_2_) with enhanced antibacterial and excellent mechanical properties was prepared *via* dopamine as a graft bridge. The microscopic morphology results show that the nanospheres are uniformly dispersed on the surface of the catheter. The physicochemical characterization confirmed that PTFE-SiO_2_ had reliable bending resistance properties, superhydrophobicity, and cytocompatibility and could inhibit thrombosis. Antibacterial results revealed that PTFE-SiO_2_ could hinder the reproduction of *E. coli* and *S. aureus*. This research demonstrates the hydroxyl-rich materials obtained by hydroboration oxidation have the advantages of better dispersion of functional coatings, indicating their potential for helpful modification of catheters.

## 1 Introduction

Long-term intravenous fluid rehydration, central venous pressure and arterial monitoring, hemodialysis, and ECMO are important treatment methods in the critical care department through the placement of central venous catheters, hemodialysis catheters, and radial artery catheters (Gilbert et al., 2013; Shimizu et al., 2020; Schalk et al., 2022). However, catheter-related infection is a common and severe complication after implantation, which often requires removal of the catheter, performing catheter end and blood culture to determine the source of infection and bacterial species (Ferreira et al., 2020; Brescia et al., 2021). Finally, antibiotics are administered, and the catheter is reinserted at another site. The infectious bacteria associated with the catheter is MASA. Due to the poor antibacterial effect of most antibiotics, the emergence of super bacteria has caused great harm to human health. This causes pain for the patient, increases the cost of treatment, and increases the risk of other complications (Akhrass et al., 2011; Jana et al., 2018).

Previous studies have shown that bacteria’s rapid, nonspecific adhesion to the hydrophilic and lipophilic groups on the catheter surface is the initial cause of catheter infection (Liu et al., 2017; Alvarenga et al., 2020; Li et al., 2020). The effect of bacterial adhesion depends on the microscopic topography and chemical properties of the catheter surface, as well as the characteristics of the bacteria (Monzillo et al., 2012; Liu et al., 2017; Yu et al., 2017; Faustino et al., 2020; Yu et al., 2021). How to reduce the colonization of bacteria in the catheter and avoid the formation of biofilm, thereby reducing the occurrence of catheter infection and the difficulty of treatment, has increasingly become a fundamental problem in clinical medicine, and more and more studies are constantly proposing new methods to solve this problem (Goncalves et al., 2014; Radu Mihu et al., 2017; Zhang et al., 2019; Gunaratnam et al., 2020). The current research on catheter-related infection globally mainly focuses on the antibacterial treatment after catheter infection. Once infected, high-grade antibiotics are required for treatment. In terms of prevention, the primary method is to coat the catheter with antibiotics. There are currently dialysis catheters that use coatings such as bismuth, which reduce bacterial colonization and thus film formation (Schindler et al., 2010; Balikci et al., 2021; Pant et al., 2022).

In addition to coating antibacterial drugs on catheters, finding the best material for antibacterial is also one of the directions we need to work on. Inspired by the origin of bacterial adhesion, we expect to develop a new type of coated vascular catheter that can effectively isolate bacterial adhesion factors. Therefore, we plan to design a novel anti-infective superhydrophobic nanocomposite-coated vascular catheter. The catheter coating and base material will not use antibacterial drugs, and the anti-infection purpose is mainly achieved by the superhydrophobicity of the surfaces of material.

## 2 Materials and methods

### 2.1 Materials

Commercial venous catheters were purchased from Arrow International, Inc. (Cleveland, United States). Both PTFE membranes (FP301350, with 0.25 mm thickness) and PTFE catheters (with 0.6 mm wall thickness and 1.0 mm inner diamater) were purchased from Goodfellow Tading Co., Ltd. (Shanghai, China). SiO_2_ nanosphere powder (average particle size is 20 nm) were purchased from Suzhou Vmicronano New Materials Co., Ltd. (Suzhou, China). Benzoin (purity ≥ 99.5%), potassium tert-butoxide (purity ≥ 99.9%), and borane tetrahydrofuran (purity ≥ 99.99%) were purchased from Adamas Reagent Co., Ltd. (Shanghai, China). Dimethyl sulfoxide (DMSO), tetrahydrofuran (THF), anhydrous ethanol (EtOH, purity ≥ 99.99%) were obtained from Sinopharm Chemical Reagent Co., Ltd. (Shanghai, China). Other reagents and materials are not treated before use unless otherwise specified.

### 2.2 Surface modification of PTFE membrane and catheter

#### 2.2.1 Preparation of PTFE-OH membrane and catheter

The circular PEFE membranes with a diameter of 14 mm and PTFE catheters with 0.6 mm thickness and 1.0 mm inner diamater (uniformly named PTFE) were ultrasonically cleaned in 75% medical alcohol for 30 min, and then freeze-dried in vacuum for subsequent experiments, respectively.

Firstly, 1.5 g of benzoin and 5.1 g of potassium tert-butoxide were dissolved in 10 and 50 ml of dimethyl sulfoxide, respectively. Then, the above two harvested solutions were mixed until present clear and homogeneous (Costello and McCarthy, 1984). Secondly, The cleaned PTFE was added to the mixed solution, and the reaction was continued for 36 h at 54°C in a constant temperature water bath. After the reaction was over, the modified PTFE were washed twice with tetrahydrofuran reagent, following washed with deionized water for 5 times. Finally, the modified PTFE were freeze-dried for 48 h, namely PTFE-M.

Finally, hydroxyl groups were introduced on the surface of PTFE according to the hydroboration oxidation reaction (Meng et al., 2015; Dan et al., 2020): PTFE-M was added to 10 ml borane tetrahydrofuran solution in three-necked flask. Then the three-necked flask was sealed, and the reaction was carried out at room temperature for 12 h. The borane tetrahydrofuran solution was then transferred out, and then 10 ml of deionized water, 10 ml of 3 mol/L NaOH solution and 10 ml of 30% H_2_O_2_ were added in sequence. Finally, place the three-necked flask into the prepared ice-water mixture and ice-bath for 3 h under a magnetic stirrer. After the reaction is completed, the modified PTFE is washed with dilute hydrochloric acid solution, alkene sodium hydroxide solution and deionized water in sequence. Finally, the hydroxylated PTFE were vacuumed in a desiccator for 48 h to remove redisual solvent and namely PTFE-OH.

#### 2.2.2 Preparation of PTFE-SiO_2_ membrane and catheter

First, 200 mg of dopamine and 2 g of nanospheres were added to a Tris (pH = 8.5) solution, which were prepared using 200 ml deionized water. Then PTFE-OH was added to the above solution which was mixed well and soaked for 4 h. Finally, the modified PTFE were washed thoroughly with deionized water, following freeze-dried in a vacuum freeze dryer for 48 h to remove redisual solvent and namely PTFE-SiO_2_.

### 2.3 Characterization and tests of prepared membrane or catheter

The morphology and surface structure of modified PTFE were carried out using a scanning electron microscope (SEM, Phenom XL, Netherlands) operating with sputter gold plating for 35 s at 5 mA at an accelerating voltage of 10 kV. FTIR spectra of modified PTFE were analyzed on a Nicolet 6700 spectrometer (Thermo Fisher, United States) in the spectrum range of 500 and 4000 cm^−1^ with a resolution of 1 cm^−1^, setting 30 scans for a single analysis. A contact angle measuring device (JC 2000D 2A, Shanghai Zhongchen Digital Technology Equipment Co., Ltd., China) was used to testing the wettability of modified PTFE. In this testing, 0.02 ml deionized water was added to the sample, and three groups of parallel samples were taken from each group of PTFE to test the water contact angle and calculate the average value.

High-precision mechanical property testing machine (HY-025CS, Shanghai Hengyu Instrument Co., Ltd., China) with a transducer with a load range of 0–1000 N was employed to testing the compressive performance of prepared catheter in dry conditions at room temperature. Catheter with a size of 2 mm inner diameter, 50 mm length, and approximately 0.5 mm in wall thickness were used for radial compression testing. The cyclic compression tests at 50% of the outer diameter of catheters were repeatedly performed 1000 times at a fixed rate of 0.2 mm/min. Each test was repeated five times during mechanical analysis.

### 2.4 Cell culture and cytocompatibility evaluation

Mice fibroblast cells (L929) were cultured with growth medium consisting of dulbecco’s modified eagle medium (DMEM), 10% fetal bovine serum and 1% penicillin/streptomycin. Before cell seeding, the commercial membranes, PTFE, and PTFE-SiO_2_ membranes were cut into discs with a diameter of 14 mm, then placed into 24-well plates one by one, following covered with sterilized stainless rings, respectively. Each sample was sterilized in 75% ethanol for 12 h and then washed with PBS three times, finally irradiated with ultraviolet for 2 h and socked in growth medium for incubation overnight. HUVECs were seeded at a density of 1.5 × 10^4^ cells/well, and the culture medium was maintained to be replaced every 2 days.

The cell viability of L929 was tested by using the Cell Counting Kit-8 (CCK-8). The cells were cultured in commercial membranes, PTFE, and PTFE-SiO_2_ membranes for 1 day, 3 days, and 5 days, respectively. When the set culture time point, the well plate was taken from the CO_2_ incubator, and then a series of operations as removal medium, wash samples in well with PBS, adding 360 μL DMEM medium and 40 μL CCK-8 solution into the lucifugal well plate in sequence. After 1 h incubation in CO_2_ incubator, The optical density (OD) value was measured with a microplate reader at a wavelength of 450 nm.

Before the cells SEM images test, the cultured L929 were fixed with 4% paraformaldehyde and dehydrated by gradient ethanol (30%, 50%, 70%, 80%, 90%, 95%, and 100%) on the 3rd day and the morphology carried out using a scanning electron microscope (SEM, Phenom XL, Netherlands) operating with sputter gold plating for 35 s at 5 mA at an accelerating voltage of 10 kV, respectively.

Additionally, the live cells (green) and dead cells (red) were stained with Calcein-AM/PI after 3 days’ culture, respectively. The dyed samples were observed immediately under the TS100 fluorescence microscope (Nikon, Japan).

### 2.5 Blood compatibility test *in vitro*

Fresh blood, which was drawn from the marginal vein, and 3.2% sodium citrate solution in a volume ratio of 9:1 (v/v) were collected using a plastic vacuum blood collection tube (2.7 ml, Becton Dickinson, United States), containing 3.2% sodium citrate solution. All animal experimental protocols are in accordance with the policy of the Institutional Review Board for Human Investigations at School of Medicine of Shanghai Jiao Tong University. Detailed procedures are available in the Supplementary Material.

### 2.6 Testing of antibacterial activity *in vitro*

*Staphylococcus aureus* (*S. aureus*, BCRC 10451) and *Escherichia coli* (*E. coli*, BCRC 11634) were used to assess the antibacterial ability of commercial membranes, PTFE, and PTFE-SiO_2_ membranes. Antibacterial activities were investigated by agar disc diffusion assay as described previously.

Firstly, commercial membranes, PTFE, and PTFE-SiO_2_ membranes were pruned into rounded membranes with a diameter of 14 mm. Then, circular membranes were irradiated under an ultraviolet lamp for 12 h, following fumigated with ethanol overnight for thorough sterilization and stored in the bechtop for further testing or evaluation. For non-quantitative synthesis, the bacterial inhibition ring of the *E. coli* and *S. aureus* cultured on the LB agar plates was assessed. In short, *E. coli* and *S. aureus* infection fluid (200 μL) was the first diffusion into the agar plates and the experiment mats (commercial, PTFE, and PTFE-SiO_2_ circular membranes) with a diameter of 14 mm and a thickness of about 300 μm were attached to the agar plate. After incubation at 37°C for 24 h, the bacterial inhibition ring of each specimen on the plate was visually measured. The inner and outer diameters and calculated diameter distinction were analyzed for evaluating the antimicrobial activity of prepared circular nanofibrous mats.

### 2.7 Biofilm experiment of catheter *in vitro*

First, all the catheter (commerical catheter, PTFE catheter, and PTFE-SiO_2_ catheter) were cut into multiple segments with 5 mm length, following ultrasonic washed with distilled water and ethanol for 5 min, respectively. Then, all the samples were immersed into 200 μL of selected medium (BHI for VRE and TSB supplemented with 1% glucose for *S. aureus*) in a 96-well plate after vacuum freeze drying for 48 h. 1.0 × 10^6^ CFU/ml bacteria suspension was made by diluting in 10 mM PBS with pH = 7.4 from overnight culture. 8 μL of the above prepared diluted bacteria suspension was added to each well, following the plate was incubated at 37°C for 24 h (Zhou et al., 2017). All the above samples were then removed and washed gently thrice with PBS to remove unattached bacteria. Then, all the samples were fixed with 4% paraformaldehyde and dehydrated by gradient ethanol (10%, 30%, 50%, 60%, 75%, 80%, 90%, and 100%) and the morphology carried out using a scanning electron microscope (SEM, Phenom XL, Netherlands) operating with sputter gold plating for 35 s at 5 mA at an accelerating voltage of 10 kV, respectively.

### 2.8 Statistical analysis

Origin 9.0 statistical software (Origin Lab Inc., United States), one-way ANOVA and Tukey’s test were applied to statistical analysis of the data. All data are expressed as mean ± standard deviation (Mean ± SD). *p*-values < 0.05 (*) were deemed to be statistically significant. * indicates *p* < 0.05, ** indicates *p* < 0.01, *** indicates *p* < 0.001.

## 3 Results and discussion

### 3.1 Microstructure and surface phychemical properties of catheter

**FIGURE 1:**
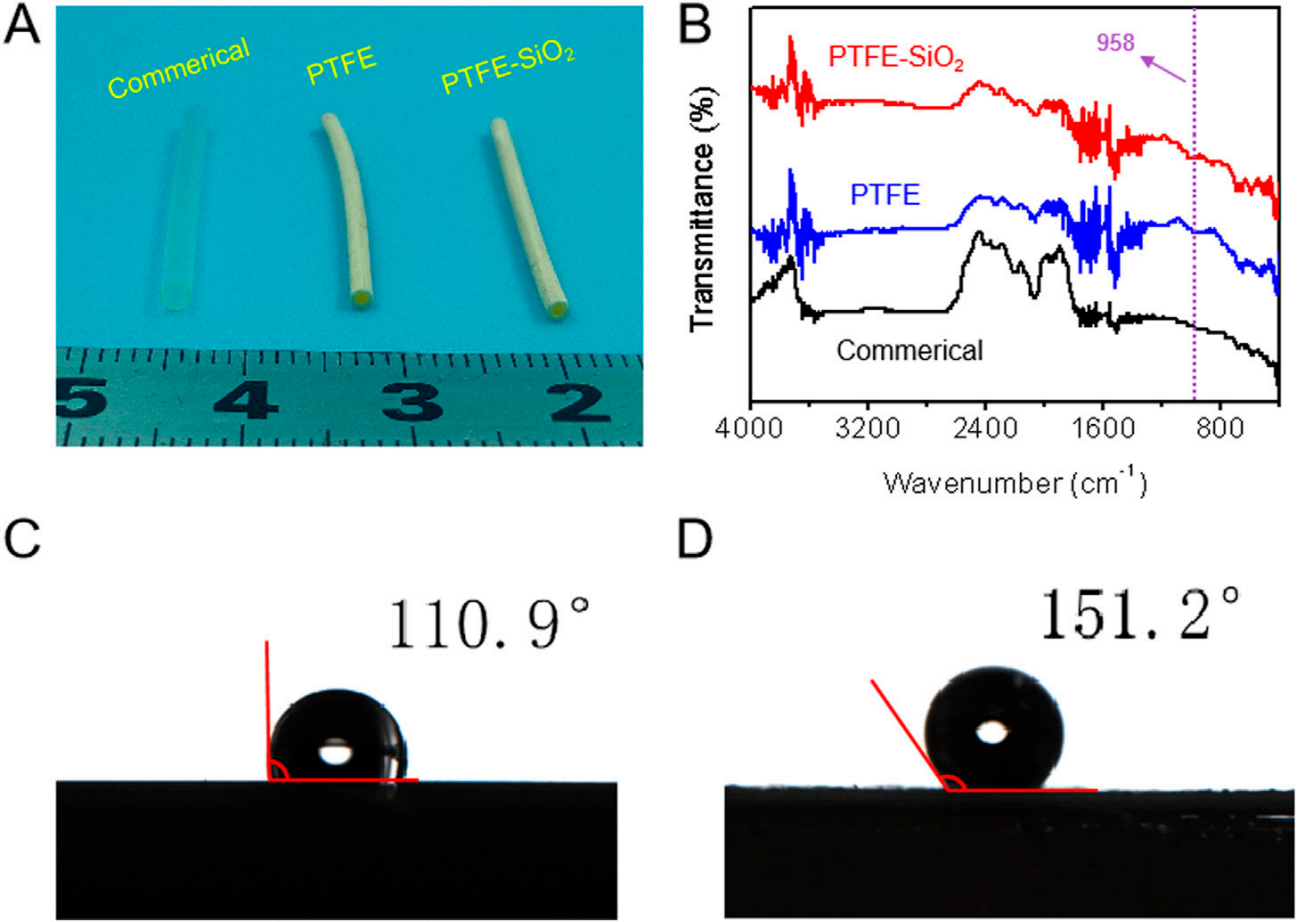
**(A)** The digital photos of commerical catheter, PTFE catheter, and PTFE-SiO_2_ catheter, respectively; **(B)** FTIR spectra of commerical catheter, PTFE catheter, and PTFE-SiO_2_ catheter, respectively; **(C)** and **(D)** Water contact angle of the lumen surface of the inner surfaces of PTFE catheter, and PTFE-SiO_2_ catheter at the 5 s time point, respectively.

As a conduit material, PTFE has advantages in mechanical properties. However, PTFE is an inert material, which limits its application in medical catheters. In order to change the chemical inertness of the surface of PTFE catheter and improve the biocompatibility of PTFE, a very active unsaturated carbon chain layer was formed on the surface of PTFE by treating the mixed solution of benzoin and potassium tert butoxide. Then, a large number of hydroxyl groups were reactive grafted on the surface of PTFE membrane by borohydride oxidation reaction, following grafted SiO_2_ nanosphere on the surface of PTFE matrix through dopamine bridging, which presented strong adhesion between the hydroxyl groups of polydopamine and the SiO_2_ nanosphere surface. Macroscopically, the catheter could maintain its tubular shape after being treatment with organic solution (Figure 1A). We obtained catheters with a length of 25 mm and an inner diameter of 1.5 mm (Figure 1A). The presence of as coated SiO_2_ nanosphere was verified *via* FTIR spectra, by which we demonstrated that PTFE-SiO_2_ catheter received more characteristic peak of stretching vibration of Si-O bond at 958 cm^−1^ after coating (Figure 1B).

With the increasing attention of superhydrophobic surfaces, the reduction of bacterial adhesion by superhydrophobic surfaces has also been considered as an effective antibacterial method (Ozkan et al., 2021; Li et al., 2022). The superhydrophobic surface interface of PTFE catheter play an important role in the adsorption and the adhesion of bacteria. As shown in Figures 1C, D, the water contact angle values of the lumen surface of the inner surfaces of PTFE catheter and PTFE-SiO_2_ catheter were 110.9° ± 1.8° and 151.2° ± 1.5° at 5 s, respectively. The SiO_2_ nanosphere-coated PTFE catheter exhibits stronger hydrophobicity. The antibacterial mechanism of superhydrophobic surfaces is that bacteria are more likely to adhere to surfaces with high surface energy, while superhydrophobic surfaces have lower surface energy, which is not conducive to the adhesion of bacteria (Boinovich et al., 2018; Huang et al., 2018; Zhan et al., 2021). Moreover, the air layer trapped by the micro-nanostructure will isolate the bacteria from the SiO_2_ coating. When the superhydrophobic catheter is in contact with bacteria, the contact area between the coating and the colony will reducing due to the effect of the air layer, which can avoid the formation of biofilm on the surface of the catheter, and finally achieve the effect of bacteriostasis.

**FIGURE 2:**
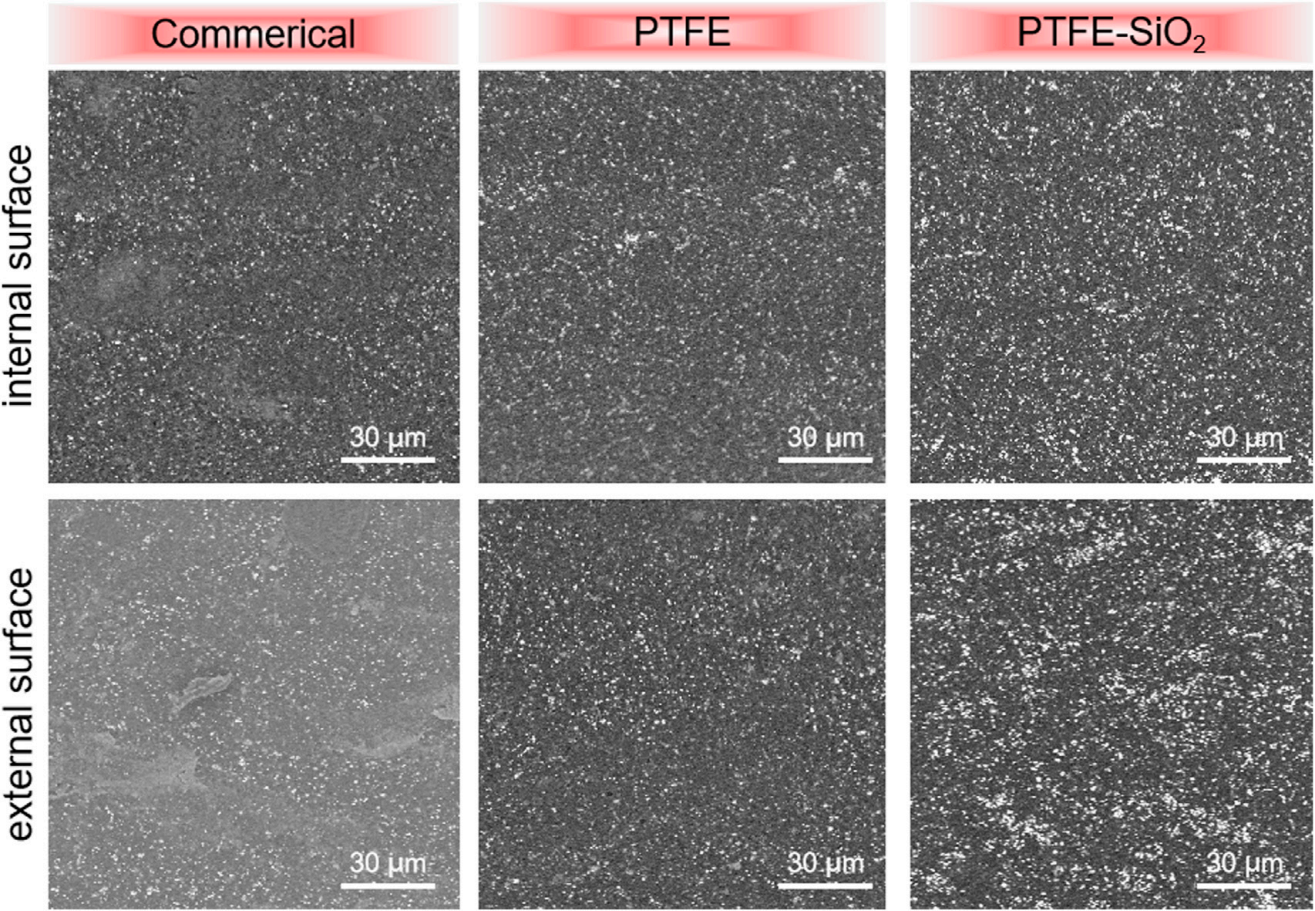
The suefaces SEM images of commerical catheter, PTFE catheter, and PTFE-SiO_2_ catheter, respectively.

The dispersion of the SiO_2_ coating is also one of the main factors affecting the antibacterial effect of the catheter surface. The scanning electron microscope images in Figure 2 showed that the coating consists of many micro- and nano-scale protrusions and pits. These micro-nano structures are mainly formed by the accumulation of nano-SiO_2_ particles. The nano-SiO_2_ is tightly fixed on the surface of the PTFE substrate by the dopamine matrix, which improves the mechanical stability of the surface of the superhydrophobic coating. Comparing the commerical group and the PTFE group, it is not difficult to find that the immobilized SiO_2_ using dopamine after hydroxylation treatment not only shows a higher graft density, but also disperses more uniformly on the PTFE surface.

### 3.2 Mechanical properties

**FIGURE 3:**
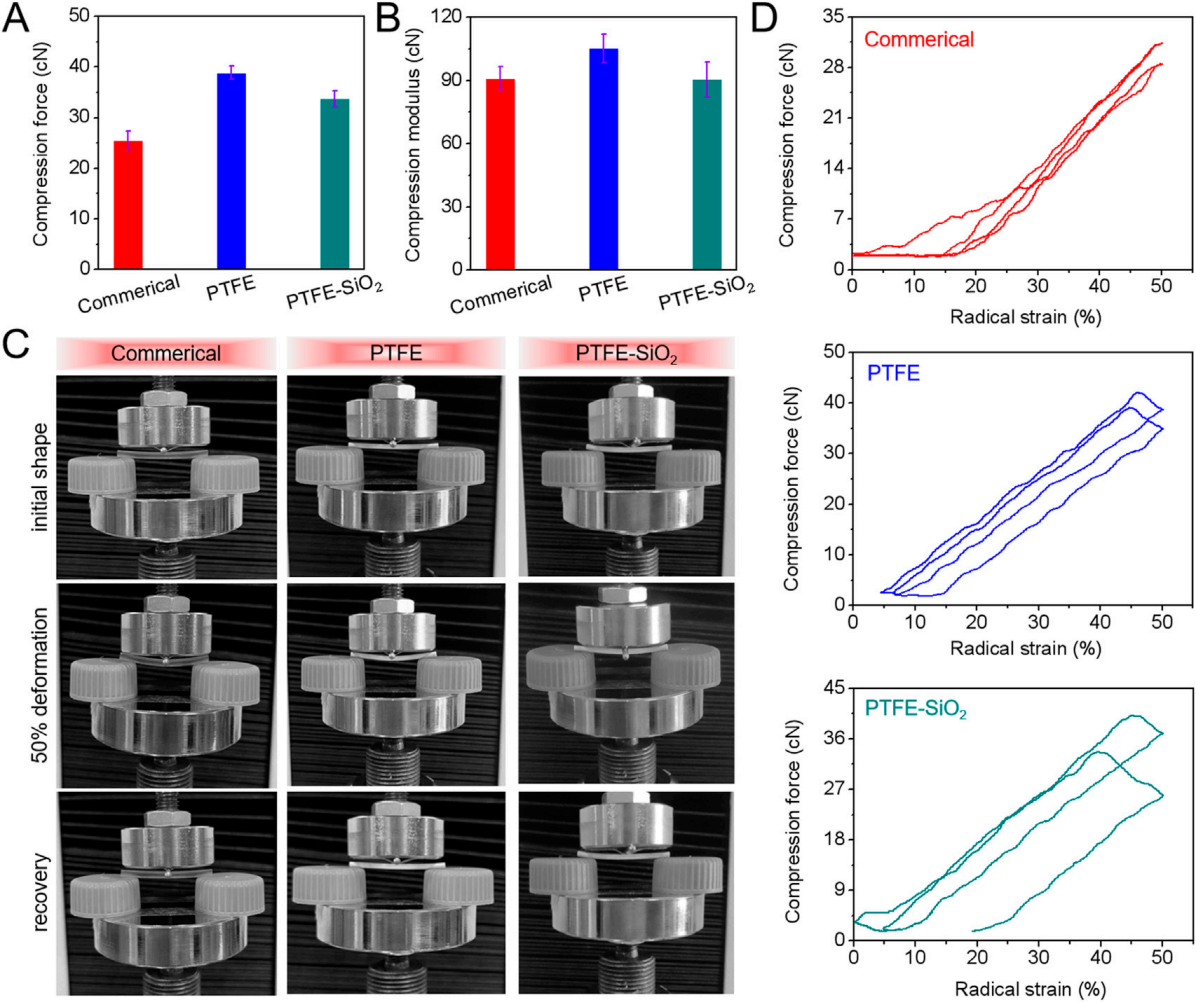
**(A)** Maximum compressive force and **(B)** compressive modulus after 1000 cycle compressive fatigue tests; **(C)** Images of three-point bending tests; **(D)** Typical the first and one thousandth loading-unloading compression cycle curves of commerical catheter, PTFE catheter, and PTFE-SiO_2_ catheter.

In order to clarify whether the original mechanical properties of PTFE catheters were changed after the grafting of SiO_2_ nanospheres, mechanical properties experiments were carried out. The results of radial cyclic compression at 50% of the outer diameter of catheters after 1000 cycle compressive fatigue tests are shown in Figure 3. As shown in Figures 3A,B, the compressive and compressive modulus after 1000 cycle compressive fatigue tests of PTFE-SiO_2_ catheter were significantly lower than those of PTFE group, but consistently superior to the commerical group. According to the standard guidelines of three-point bending test of ASTM F2606-09 as present in Figures 3C, D, all the samples were bendable and returned to their original shape without any visible damage and permanent deformation. Therefore, the modified catheter still maintains reliable bending resistance, which will be sufficient to support the catheter against any possible external force during surgical operation.

### 3.3 Cytocompatibility and hemocompatibility *in vitro*

**FIGURE 4:**
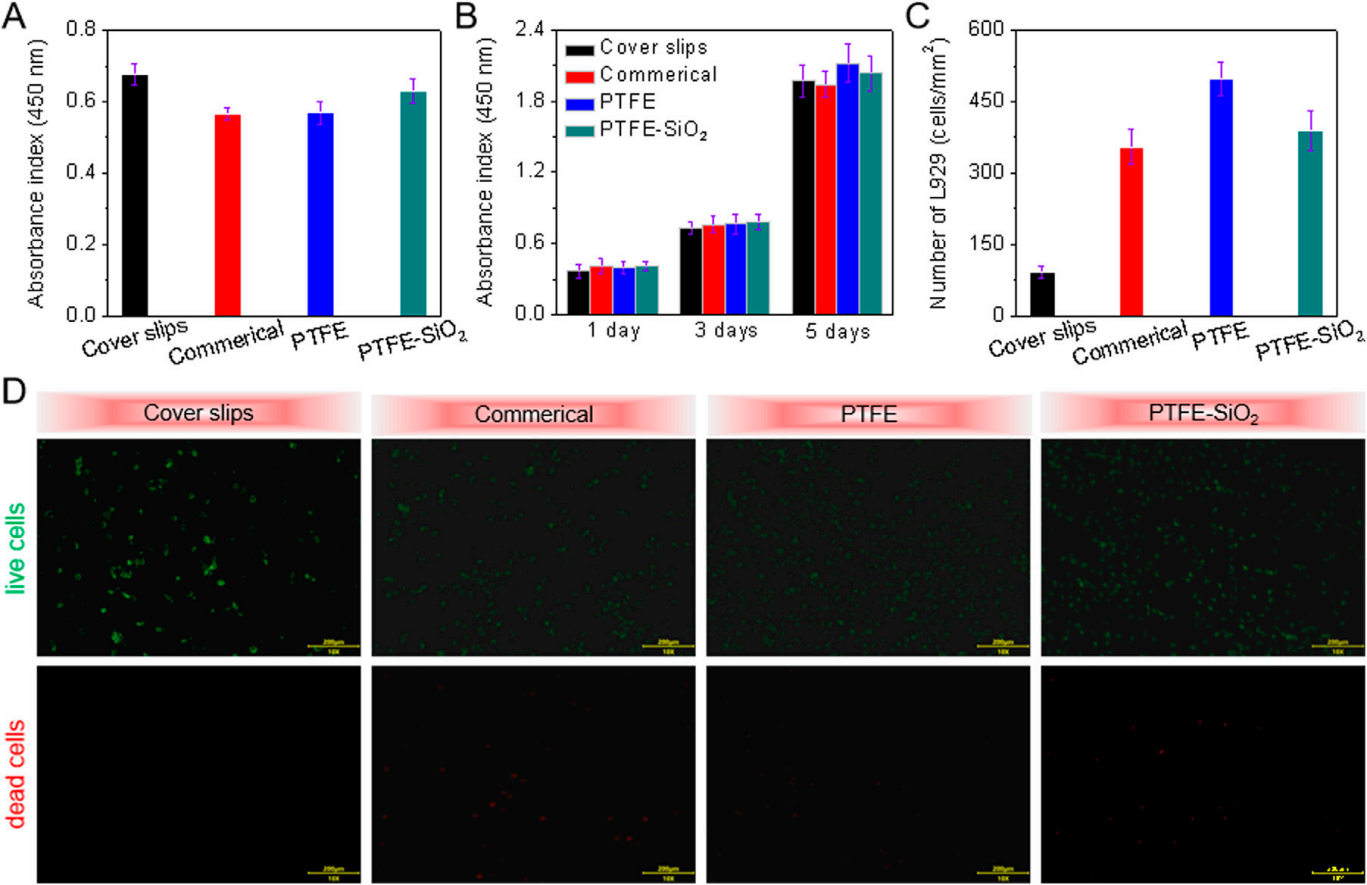
CCK-8 assay of **(A)** the adhesion viability after 24 h culture and **(B)** proliferation viability after 1, 3, and 5 days culture of HUVECs onto cover slips, commerical catheter, PTFE catheter, and PTFE-SiO_2_ catheter, respectively; **(C)** Quantitative number measurements of L929 cells on the inner surfaces of cover slips, commerical catheter, PTFE catheter, and PTFE-SiO_2_ catheter after culturing for 3 days; **(D)** Live/dead staining viability assay of L929 cells cultured on the inner surfaces of cover slips, commerical catheter, PTFE catheter, and PTFE-SiO_2_ catheter after culturing for 3 days.

To test the biocompatibility of the samples before and after modification, the same amount of L929 was inoculated on each sample. As shown in Figures 4A,B, the adhesion ability of the PTFE-SiO_2_ group to L929 was higher than that of the commercial group and the PTFE group but still lower than that of the cover slips group. Figures 4C,D show that by day 3, the number of viable cells in the cover slips group and the commerical group was less than that in the other two groups. The PTFE-SiO_2_ group had the smallest viable cells, mainly due to the hydrophobic surface and chemical inertness caused by the fluorocarbon structure. The SiO_2_ nanocluster structure is unsuitable for cell adhesion and proliferation.

After vascular puncture, the catheter stays in the body for a long time, which can easily lead to nonspecific protein adsorption, microbial infection, and blood coagulation (Hitz et al., 2012; Erathodiyil et al., 2020). These phenomena eventually lead to deep vein thrombosis (PICC-DVT), pulmonary embolism (PE) due to thrombus shedding, and catheter-related bloodstream infections (CRBSIs) due to microbial infections. Currently, drugs such as heparin and antibiotics are injected into patients to reduce catheter-related complications. However, neither heparin nor antibiotics maintain long-term efficacy in the body. Repeated injections of chemicals will not only reduce the patient’s immunity and cause suffering from drug side effects. Therefore, optimizing and modifying existing materials is an effective measure to reduce catheter-related complications and improve the anticoagulant and antibacterial properties of catheters.

**FIGURE 5:**
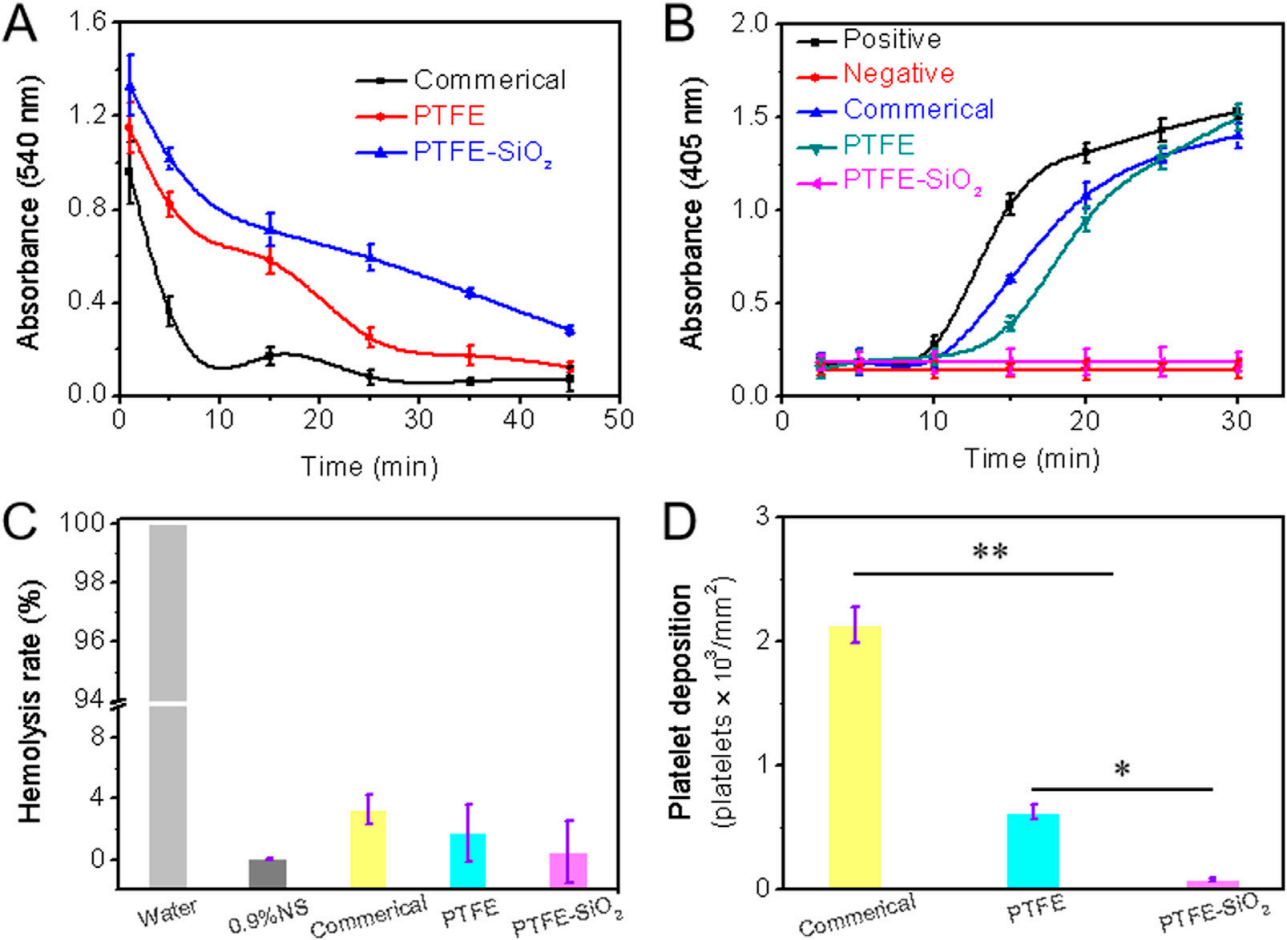
**(A)** Whole blood clotting time **(B)** Plasma recalcification time; **(C)** Quantification of relative hemolysis rate; **(D)** Platelet deposition determined by lactate dehydrogenase assay. (For plasma recalcification time test, TCPs exposed to PPP with and without CaCl_2_ were used as positive control and negative control, respectively; For hemolysis test, water and 0.9% normal saline (NS) serve as positive and negative groups, respectively; n = 5, *p < *0.05*, **p < *0.01*).

The anticoagulant property of all samples could be evaluated directly by whole blood clotting time, plasma recalcification time, quantification of relative hemolysis rate, and quantification of lactate dehydrogenase activity. We assessed the whole blood clotting of catheter by the whole blood clotting time test. After the whole blood incubation, the absorbance of the supernatant was measured at 540 nm, as shown in Figure 5A. Compared with the other two groups, the PTFE catheter coated with SiO_2_ nanospheres had the maximum absorbance at any time point. The higher the absorbance, the higher the hemoglobin content in the supernatant, which means that there are more blood cells in the free state, less blood cells that produce coagulation, and the slower the coagulation speed. All in all, under the same experimental conditions, the clotting time of the control group was significantly shorter, indicating that the coated-modified PTFE catheter has highly hydrophobic properties and can resist the adhesion of proteins to the material surface. Therefore, PTFE-SiO_2_ catheter can reduce the adhesion of blood cells and has outstanding anticoagulant properties.

As we all know, the essence of blood coagulation is the process of fibrinogen in plasma from soluble to insoluble, and blood coagulation is divided into two types: intrinsic coagulation and extrinsic coagulation. All coagulation factors involved in intrinsic coagulation are provided by plasma. When the plasma comes into contact with the material, the electrical charge on the surface activates the coagulation factors in the plasma. The plasma recalcification curve is a method used to characterize the endogenous coagulation system. The recalcification time is the time it takes for the plasma to coagulate after removing the calcium source and then adding Ca^2+^. The kinetics of plasma recalcification can be seen in Figure 5B for the three water conduits, the positive control group and the negative control group. The positive control has the fastest clot formation time at 15 min, while the clot formation time of the commerical and PTFE group were 20 and 24 min, respectively. Notably, the PTFE-SiO_2_ group consistently kept a low absorbance and closed to the negative control without emerging the inflection point. The above results indicate that the PTFE-SiO_2_ group has a superhydrophobic surface interface effect, so the activation of endogenous coagulation is less, and the anticoagulation performance of the catheter is improved.

The hemolysis rate of a material refers to the degree to which hemoglobin is lysed by erythrocytes after the material is in contact with erythrocytes. According to international standards, the hemolysis rate of biological materials should be less than 5% (Zhu et al., 2021). Figure 5C is the hemolysis rate of PTFE catheters before and after coating. As shown in the figure, the hemolysis rate of the uncoated PTFE catheter was 1.756%, while after the SiO_2_ nanosphere coating, the anti-hemolysis ability was significantly reduced. In conclusion, PTFE-SiO_2_ had the best blood compatibility among all samples, and the hemolysis rate was only 0.521%, which was almost close to that of the negative control group.

The lactate dehydrogenase (LDH) activity of platelet adhesion on the inner surface of catheter after 2 h incubation with rabbit platelet-rich plasma are shown in Figure 5D. The deposition of adhered platelets on the inner surface of PTFE-SiO_2_ catheter was 85 platelets/mm^2^, which was significantly reduced to 622 platelets/mm^2^ for the PTFE catheter and to 2136 platelets/mm^2^ for the commerical catheter. SiO_2_ nanosphere coated-modified PTFE catheter adsorbed fewer platelets compared with the PTFE catheter, indicating that coated SiO_2_ nanosphere not only significantly reduced platelet deposition but also efficiently suppressed the activation and transmutation of platelets.

### 3.4 Antibacterial effect *in vitro*

After the catheter enters the blood vessel, some nonspecific proteins (polyglycan matrix, *etc.*) will form a thin film on the surface of the catheter. Bacteria will then stick to the film and increase, following a biofilm formed. Because the resulting biofilm is not recognized by tissues and the bacteria are not killed by the immune system, it can eventually lead to bacterial infections. The superhydrophobic surface is an effective method to limit the formation of biofilm, which can inhibit the adhesion of bacteria to the material surface.

**FIGURE 6:**
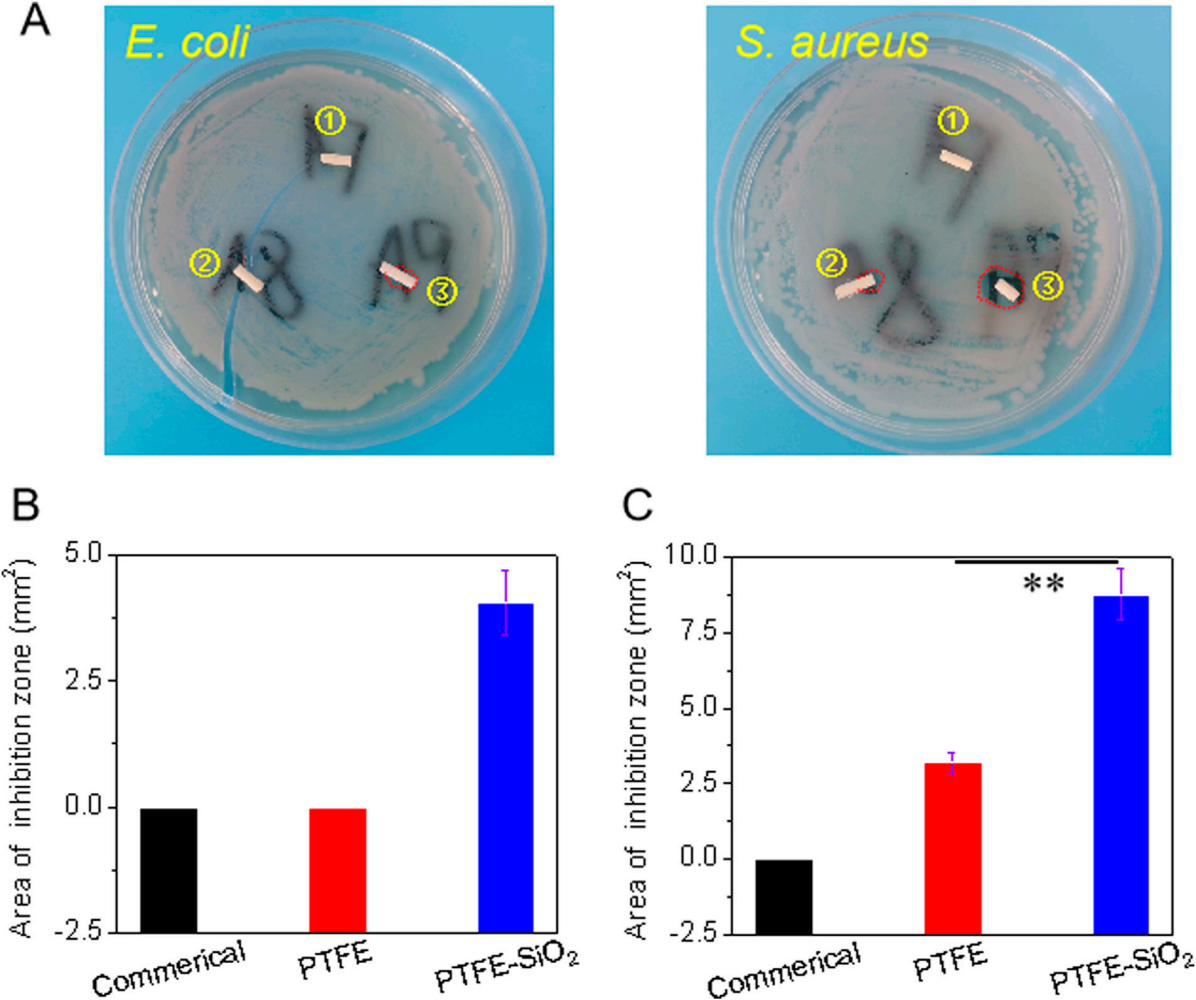
**(A)** Visual images of the antibacterial zone of catheter to *Escherichia coli* (*E. coli*) and *Staphylococcus aureus* (*S. aureus*) after incubation for 6 h. Spot ①, ②, and ③ represents commerical catheter, PTFE catheter, and PTFE-SiO_2_ catheter, respectively; of commerical catheter, PTFE catheter, and PTFE-SiO_2_ catheter, respectively; **(B)** and **(C)** Quantified antibacterial zone of commerical catheter, PTFE catheter, and PTFE-SiO_2_ catheter after 6 h incubation with *E. coli* and *S. aureus*, respectively. (n = 5, **p < *0.01*)

The bactericidal effect on Gram-negative bacteria (*Escherichia coli*, *E. coli*) and Gram-positive bacteria (*Staphylococcus aureus*, *S. aureus*) was evaluated to determine whether the superhydrophobic SiO_2_ nanosphere coating had a pronounced antibacterial effect. As shown in Figure 6, after co-culture of PTFE-SiO_2_ catheters on the solid medium of two bacteria, bacteriostatic circles appeared, and the areas of bacteriostatic processes were 4.05 and 8.75 mm^2^, respectively. Compared the antibacterial effects of the three groups of samples, it can be found that the commercial group samples did not show evident antibacterial rings after co-culture on the two kinds of colony media, and the PTFE catheter only showed small antibacterial rings on the surface of the medium with *S. aureus*. These results indicate that the superhydrophobic conduit coated with SiO_2_ nanospheres has specific antibacterial properties.

**FIGURE 7:**
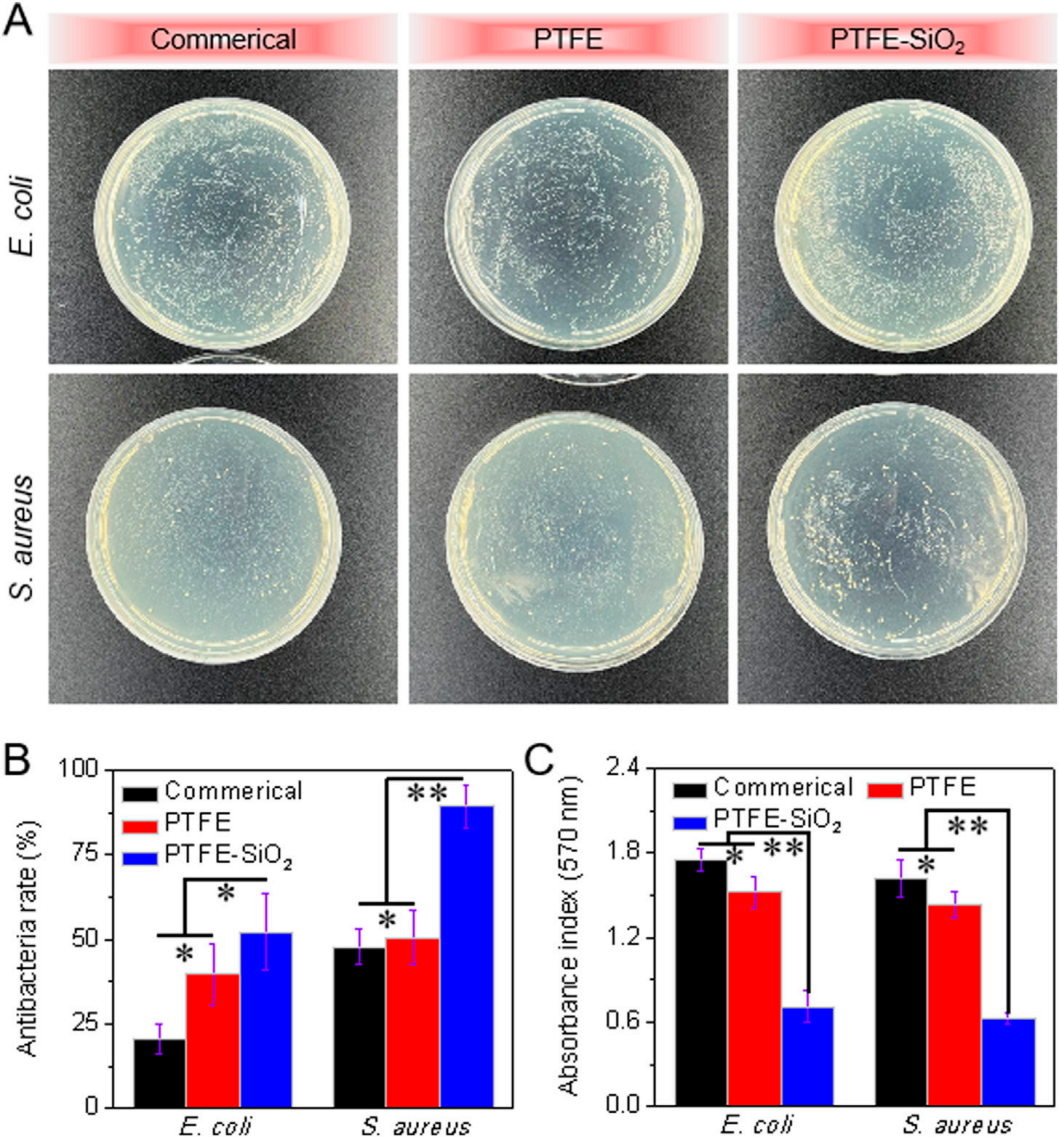
Quantitative analysis of the antibacterial properties of catheter: **(A)** Conoly-counting assay of catheter against *E. coli* and *S. aureus*; **(B)** Antibacterial rate of catheter after 6 h incubation with *E. coli* and *S. aureus* (quantitative analysis of colony statistics); **(C)** Quantitative analysis of photometric method after 6 h incubation with *E. coli* and *S. aureus*. (n = 5, *p < *0.05*, **p < *0.01*)

We also compared the bacteriostatic rate of the three groups of samples by colony statistical test. As shown in Figure 7A, *E. coli* and *S. aureus* were cultured for 6 h, respectively. Then, a certain amount of bacterial liquid was drawed and placed in a sterile centrifuge. After adding the three samples to the corresponding centrifuge tubes, the centrifuge tubes were placed in a constant temperature shaker for co-cultivation for a certain period of time, and the bacterial solution was diluted 10,000 times. Then take 10 μL of the diluted bacterial culture solution, spread it evenly on the surface of the petri dish containing the solid medium in one direction, place it in a constant temperature shaker for 6 h, and count the number of colonies. After superhydrophobic modification using SiO_2_ nanospheres, the antibacterial rate of the catheter against *E. coli* was enhanced from 39.7% to 52.1%, and the antibacterial rate against *S. aureus* was increased from 50.5% to 89.3% (as shown in Figure 7B). It shows that the coating using SiO_2_ nanospheres enhances the antibacterial activity of the PTFE catheter, which may due to the superhydrophobic coating of SiO_2_ nanospheres not being conducive to the formation of the bacterial film.

To illustrate that the modified PTFE catheter has prominent antibacterial properties, the antibacterial properties were quantitatively analyzed by photometry. As shown in Figure 7C, after co-culturing the catheter with the bacterial solution for 6 h, the absorbance of the commerical group and the PTFE group was significantly higher than that of the PTFE-SiO_2_ group, regardless of whether it was *E. coli* and *S. aureus*. It shows that the catheter of the commercial group does not have antibacterial properties, which is consistent with the inhibition zone and colony statistics results. In conclusion, the antibacterial ability of the PTFE catheter was significantly improved after the layer modification.

**FIGURE 8:**
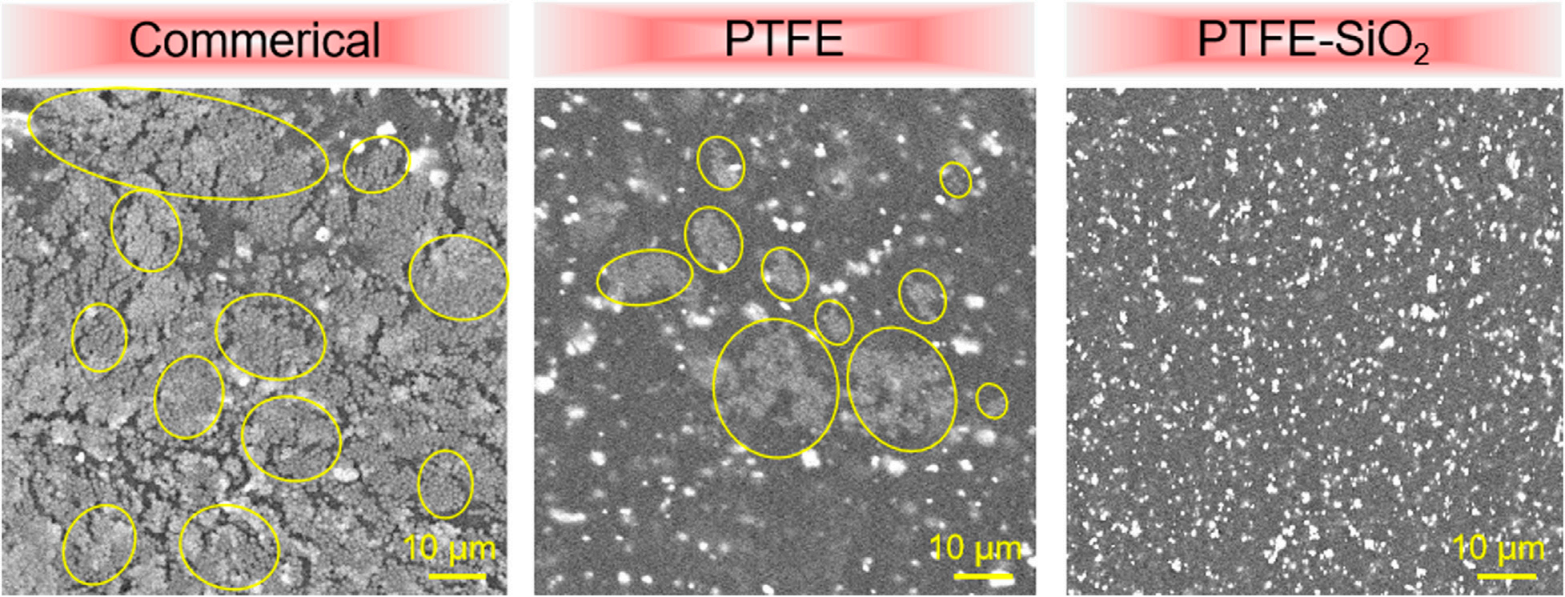
SEM images of *S. aureus* on the surfaces of commerical catheter, PTFE catheter, and PTFE-SiO_2_ catheter, respectively. (Yellow solid represent bacteria in biofilm).

Surface functionalization is an effective strategy to improve the application potential of medical catheters. Proper surface wettability is the key to improve the antibacterial ability of the catheter (Chauhan et al., 2014). The mechanism of antibacterial activity of SiO_2_ nanosphere coated tough catheter against *S. aureus* was examined by SEM images. As in Figure 8, SEM images showed large numbers of *S. aureus* on the suefaces of commerical catheter. On the surface of PTFE catheter, SEM images show the number of viable *S. aureus* to be greatly reduced, confirming the anti-*S. aureus* nature of the surface of electronegativity of PTFE. It is worth noting that although PTFE-SiO_2_ catheter has a large contact angle, it also prevents the formation of *S. aureus* biofilm, possibly because of the large surface anionic charge of SiO_2_, which were uniformly distributed on the PTFE surface.

## 4 Conclusion

In the present study, we developed a SiO_2_ nanosphere-coated PTFE catheter (PTFE-SiO_2_) based on hydroxyl-rich materials obtained by hydroboration oxidation. SEM, FTIR, water contact angle, mechanical tests, cell and blood compatibility test *in vitro* were used to analyze the chemical structure, microstructure, surface wettability, cytocompatibility, and hemocompatibility of PTFE-SiO_2_ catheter. The results show that the SiO_2_ nanospheres can be uniformly dispersed on the surface of the PTFE catheter, and the coating will not significantly change the bending resistance of the catheter. Moreover, the catheter demonstrated a safe hemolysis rate of less than 5% while also not causing coagulation. Finally, antibacterial experiments showed that PTFE-SiO_2_ catheter was the group with the highest antibacterial rate among the three groups. In conclusion, the SiO_2_ nanospheres-coated PTFE catheter with regular morphology and high bending resistance property has a potential application prospect in anticoagulant and anti-infective catheters.

## Data Availability

The original contributions presented in the study are included in the article/Supplementary Material, further inquiries can be directed to the corresponding author.
