# Supplementary material for: SiO2 nanosphere coated tough catheter with superhydrophobic surface for improving the antibacteria and hemocompatibility
Source: Front Bioeng Biotechnol. 2023 Jan 10;10:1067139. doi: 10.3389/fbioe.2022.1067139 (PMC9872198; doi:10.3389/fbioe.2022.1067139)
Supplement: Supplementary file 1 [file DataSheet1.docx]

**Supporting Information**

SiO_2_ nanosphere coated tough catheter with superhydrophobic surface for improving the antibacteria and hemocompatibility

Weixing Zhang ^a^, Juan Du ^b^, Tonghe Zhu ^b^, Ruilan Wang ^a, *^

*^a^ Department of Critical Care Medicine, Shanghai General Hospital, Shanghai Jiao Tong University, School of Medicine,* *650 Xinsongjiang Rd., Shanghai 201600, P.R. China*

*^b^ School of Chemistry and Chemical Engineering, Shanghai Engineering Research Center of Pharmaceutical Intelligent Equipment, Shanghai Frontiers Science Research Center for Druggability of Cardiovascular Non-coding RNA, Institute for Frontier Medical Technology, Shanghai University of Engineering Science, 333 Longteng Rd., Shanghai 201620, P.R. China*

1. **Supplementary experimental section**

**1.1 Whole blood clotting time test**

First, all samples were pre-incubated with PBS for 2 h in 48-well plates. Then, PBS solution was changed with 10 μL CaCl_2_ solution (0.1 M). After adding 100 μL fresh bloods to each sample, the blood clotting was activated. All samples (including tissue culture plate (TCP)) were incubated at room temperature for 1, 5, 15, 25, 35, and 45 min. At the end of each time point, 1 mL of distilled water was added to the wells for another 5 min. Finally, 100 μL solution from each well (n = 5) was transferred to a 96-well plate to measure the absorbance at 540 nm.

**1.2 Plasma recalcification test**

First, all samples were added 500 μL of PPP and incubated at 37 ℃ for 60 min in 24-well plates. Then 100 μL of platelet-poor plasma (PPP) of every well was transferred to a 96-well plate and another 100 μL of CaCl_2_ solution (0.025 M) was added into the well. The whole blood clotting kinetics was monitored by measuring the absorbance at 405 nm at 37 ℃ (every 30 s for 45 min). TCPs exposed to PPP with and without CaCl_2_ were used as positive control and negative control, respectively. Five parallel experiments were proceeded to calculate the mean absorbance at each time point.

**1.3 Hemolysis test**

Healthy red blood cells (HRBCs) were obtained according to pre-treatment procedures in our previous reports for hemolysis assay. In brief, HRBCs were obtained by centrifuging the fresh blood (1200 r/min for 10 min), followed by washing the precipitates with PBS 5 times to completely remove the serum. The HRBCs were diluted 10 times with PBS before hemolysis assay. The diluted HRBCs (0.2 mL) mixed with PBS solution (10 mL) were added into a centrifugal tube with its bottom covered with PTFE catheters (1.0 mm inner diameter, 10 mm length, 0.6 mm wall thickness). The diluted HRBCs (0.2 mL) were mixed with 10 mL Milli-Q water (abbreviated water (+), as a positive control) and 10 mL normal saline (abbreviated 0.9%NS (-)) (as a negative control) in centrifugal tubes for comparison. After a gentle shaking, all samples were incubated at 37 °C for 2 h. Then all the HRBC suspensions were taken away carefully and centrifuged at 2000 rpm/min for 5 min. The absorbance at 545 nm of the supernatant (hemoglobin) was determined by Lambda 25 UV-Vis spectrophotometer (Perkin Elmer, USA). Hemolysis rate (HR) was defined as Eq. (1):

HR (%) = (A_s_-A_n_)/(A_p_-A_n_)×100% (1)

where, A_s_, A_p_, and A_n_ stand for the absorbency of the experimental sample, the positive control and the negative control, respectively. The mean and standard deviation of the triplicate centrifugal tubes for each sample were calculated.

**1.4 Platelet adhesion test**

Platelet-rich plasma (PRP, 2×10^7^ platelets/mL) was obtained by centrifugation from Fresh New Zealand white rabbit blood at 1200 rpm for 10 min at room temperature. Round samples (diameter=14 mm) tailored from PTFE membranes were placed into 24-well plates individually and sterilized with 75% ethanol immersion for 2 h and rinsed with deionized water three times. PRP (500 µL/well) was added onto the surface of samples. After 2 h of incubation at 37 °C with mild shaking, samples were gently rinsed with deionized water to wash away non-attached platelets. Then, the platelets deposited on the surface were fixed in 4% paraformaldehyde for 2 h, then dehydrated with gradient ethanol (30%, 50%, 70%, 80%, 90%, 95%, 100%) and dried at room temperature. The platelets adhered to samples were sputter-coated with gold for SEM observation. The number of adherent platelets was determined by detecting the activity of lactate dehydrogenase (LDH Release Assay Kit, Beyotime, Nantong, China) present after cell lysis as previously described.

**1.5 Statistical analysis**

Origin 9.0 statistical software (Origin Lab Inc., USA), one-way ANOVA and Tukey’s test were applied to statistical analysis of the data. All data are expressed as mean ± standard deviation (Mean ± SD). *P*-values <0.05 (*) were deemed to be statistically significant. * indicates p < 0.05, ** indicates p<0.01, *** indicates p<0.001.
